# Pediatric rheumatology in Africa: thriving amidst challenges

**DOI:** 10.1186/s12969-021-00557-7

**Published:** 2021-05-07

**Authors:** Angela N. Migowa, Djohra Hadef, Wafa Hamdi, Oscar Mwizerwa, Madeleine Ngandeu, Yassmin Taha, Faleye Ayodele, Kate Webb, Christiaan Scott

**Affiliations:** 1 Aga Khan University Medical College East Africa, Department of Paediatrics and Child Health, 3rd Parklands Avenue, P. O Box 30270, Nairobi, Kenya; 2 Batna 2 University, Faculty of Medicine, 05000 Ezzohor city, Batna Algeria; 3 University of Tunis El Manar, Campus Universitaire Farhat Hached B.P. n° 94 Rommana Tunis, 1068 Tunis, Tunisia; 4 University of Rwanda, KG 11 Ave, Kigali, Rwanda; 5 University of Yaoundé, Boîte Postale 337, Yaoundé, Centre Region Cameroon; 6 Ahmed Gasim Children’s Hospital Khartoum, Sudan Bahri Street, Downtown, Tuti Island, Bahri, Khartoum State Sudan; 7 Lagos State University Teaching Hospital, Nigeria 1- 5 Oba Akinjobi Way, Street, Ikeja, Lagos Nigeria; 8 University of Cape Town, South Africa Rondebosch, Cape Town, 7700 South Africa

**Keywords:** Pediatric rheumatology, Africa

## Abstract

**Background:**

Pediatric Rheumatology is an orphan specialty in Africa which is gradually gaining importance across the continent.

**Main body:**

This commentary discusses the current state of affairs in the sphere of Pediatric Rheumatology across Africa and offers practical strategies to navigate the challenges encountered in research, models of care, education and training. We outline the establishment, opportunities of growth and achievements of the Pediatric Society of the African League Against Rheumatism (PAFLAR).

**Conclusion:**

This commentary lays the foundation for establishment of a formidable framework and development of partnerships for the prosperity of Pediatric Rheumatology in Africa and beyond.

## Background

**Fig. 1:**
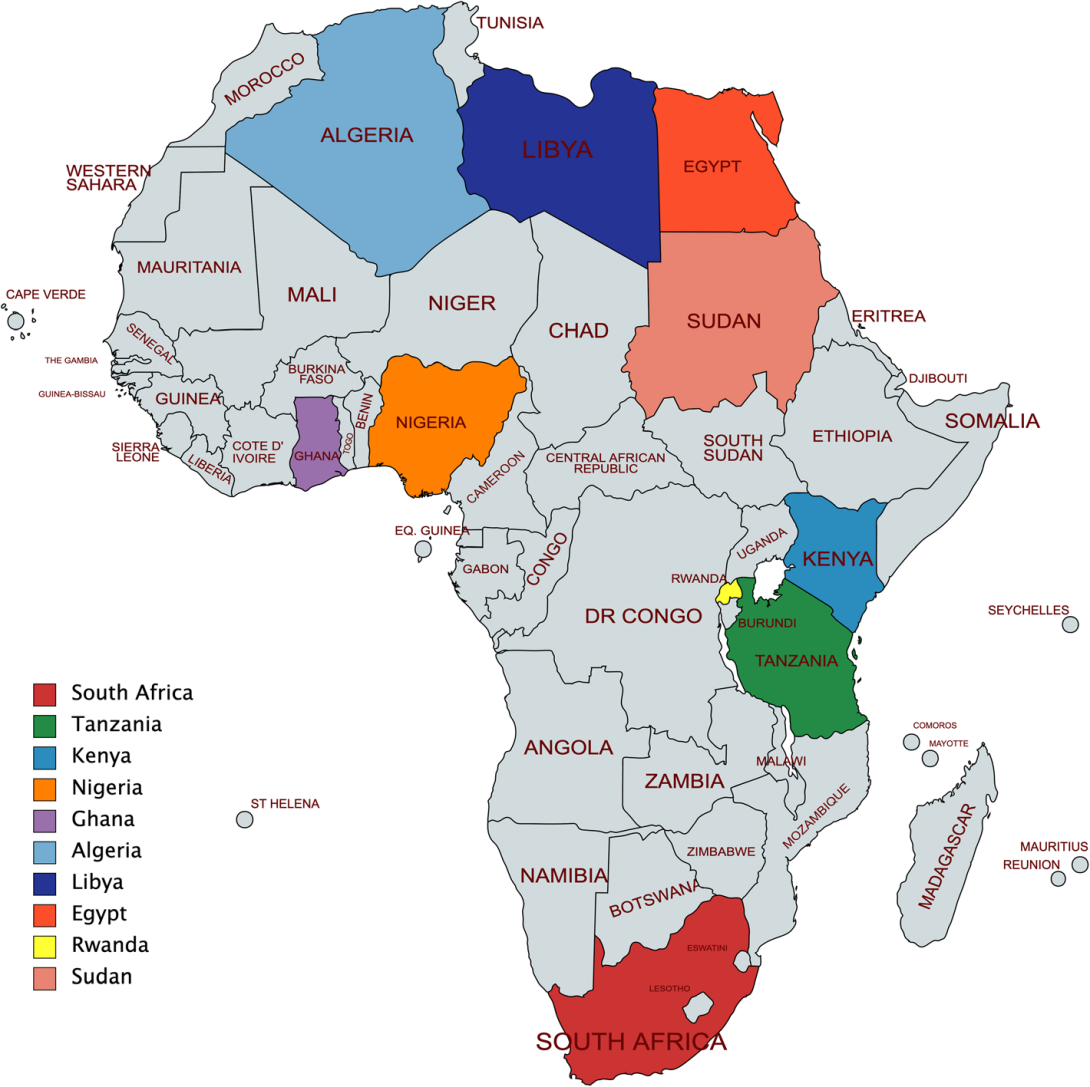
African Countries with Pediatric Rheumatology Centres

Africa is the second most populous continent in the world with an estimated 1.2 billion people and 496 million children [1, 2]. The pediatric population in Africa is postulated to rise to 661 million by 2030, becoming the continent with the most children [2]. There are approximately 6–7 million children afflicted worldwide with rheumatic disease and the majority of these, approximately 78%, live in Asia and Africa [3–5]. Rheumatic diseases often result in pain, disability, poor mental health and increased all-cause mortality [6]. Pediatric Rheumatic Diseases (PRDs) are perceived to be rare in Africa probably due to lack of local expertise and reporting [3–5]. In reality, there are significant numbers of children with rheumatic diseases in Africa who deserve care [3–5]. Acknowledging the burden of pediatric rheumatic diseases across Africa is an important critical step in providing care to these patients [7–15]. As we strive to achieve universal healthcare, non-communicable diseases such as PRDs require attention [16–18]. Developing the pediatric rheumatology workforce is a prerequisite to providing children with rheumatic diseases access to healthcare and good clinical outcomes [19]. Significant strides have been made to promote the discipline of Pediatric rheumatology in Africa as evidenced by the various centers that exist in the 10 of the 54 countries in Africa illustrated in Table 1 and Fig. 1. However much more still needs to be done to establish pediatric rheumatology services across the continent.

**Table 1:** Overview of Paediatric Rheumatology Centres in Africa

Country	Centre	Paediatric Rheumatologist	Contacts	Training Centre	Inpatients /year	Outpatient/year	Availability of Occupational Therapy/Physiotherapy	Availability of Synthetic DMARDs	Availability of Biological DMARDs
**Northern Africa Region**									
**1.Egypt**	**Abu el Reesh University Hospital**	**Hala M Lofty**	**Dr_hlofty@yahoo.com**	**Cairo University** **EULAR Courses**	**600**	**8000**	**Available**	**Available**	**Ant TNF** **Anti IL1** **Anti IL6** **Anti IL17** **Anti IL23** **JAK Inhibitors** **Anti CD 20**
**2.Libya**	**Benghazi Children Hospital**	**Halima Mohamed Benamer**	**benamer_h@yahoo,co.uk**	**Benghazi Children Hospital**	**50**	**300**	**Available**	**Available**	**Ant TNF** **Anti IL1** **Anti IL6** **Anti CD 20**
	**Tripoli Children’s Hospital**	**1.Soad Hashad** **2.Hala Etayari** **3.Eman Elmisllati-Traineee** **4.Magda Tofil-Trainee** **5.Zohairah Awhidah-Trainee** **6.Aya Twati-Trainee**	**soadhashad@hotmail.com** **h.eltayari@uot.edu.ly** **dr.iman.misllati@gmail.com** **magdanaltfyl@gmail.com** **zohairah.awhaidah11@gmail.com**	**Tripoli Children’s Hospital** **Instituto Giannina Gaslini, Genoa/Italy** **EULAR Online Course**	**20**	**800**			
**3.Tunisia**	**-Kassab Institute / Manouba** **-Mongi Slim Hospital Tunis** **-Children hospital Bechir Hamza / Tunis**	**Wafa HAMDI** **Leila Souabni** **Zohra Fitouri**	**wafahamdi6@yahoo.fr**	**Paris-Descartes University FRANCE**	**150**	**600**	**Available**	**Available**	**Anti TNF (5 molecules)** **Anti IL6** **JAK Inhibitors** **Anti CD20** **Anti IL17**
**4.Algeria**	**Batna 2 University**	**Djohra HADEF**	**djohra.hadef@paflar.org**	**France**	**30**	**100**	**Available**	**Available**	**Ant TNF** **Anti IL1** **Anti IL6** **Anti IL17** **Anti CD 20**
**Eastern Africa Region**									
**5.Kenya**	**Aga Khan University Medical College East Africa**	**Angela Migowa**	**angela.migowa@** **paflar.org** **angela.migowa@aku.edu**	**McGill University Health Centre**	**30**	**200**	**Available**	**Available**	**Ant TNF** **Anti IL1** **Anti IL6** **JAK Inhibitors** **Anti CD 20**
	**University of Nairobi**	**Lawrence Owino**	**jahkaruoth2000@gmail.com**	**University of Cape Town**	**50**	**300**			
**6.Tanzania**	**Dar es Salaam**	**Francis Fredrick**	**fredrick.francis78@gmail.com**	**EULAR Online Course** **University of Cape Town**	**50**	**300**	**Available**	**Available**	**Anti CD 20** **Anti IL6**
**7.Sudan**	**Khartoum**	**Abubaker Fadlelmola**	**ytaha@doctors.org.uk**	**University of Cape Town**	**100**	**400**	**Available**	**Available**	**Inaccessible**
		**Yassmin Taha**		**United Kingdom**					
**Western Africa Region**									
**8.Nigeria**	**Lagos State University Teaching Hospital**	**Faleye Ayodele**	**faleyeayo2013@gmail.com**	**University of Cape Town**	**10–20**	**40**			**Anti TNF** **Anti CD 20**
**9.Ghana**	**Tamale**	**Yaninga Halwani Fusweni**	**yaninga@yahoo.com**	**University of Cape Town**	**6**	**5**	**Available**	**Available**	**Inaccessible**
**Southern Africa Region**									
**10.South Africa**	**University of Cape Town**	**Chris Scott**	**chris.scott@uct.ac.za**	**South Africa and Belgium**	**450**	**1500**	**Available**	**Available**	**Ant TNF** **Anti IL1** **Anti IL6** **JAK Inhibitors** **Anti CD 20**
		**Kate Webb**	**drkatewebb@gmail.com**	**South Africa and UK**					
		**Nicky Brice**		**University of Cape Town**					
		**Waheba Slamang**	**waheba.slamang@gmail.com**	**University of Cape Town**					
		**Deepthi Abraham**	**deepthi@sun.ac.za**	**Tygerberg Hospital**	**113**	**516**			
	**Johannesburg**	**Gail Faller**	**gail.faller8@live.com**	**University of Witwatersrand**	**20–40**	**200**			
		**Bhadrish Mistry**	**bjmistry@mweb.co.za**	**University of Western Australia**	**20–40**	**200**			
		**Priya Ambaram**	**priya.ambaram@gmail.com**	**University of Witwatersrand**	**4–20**	**395**			
	**Durban**	**Kogie Chinniah**	**chinniahk2@ukzn.ac.za**	**Nelson R Mandela School of Medicine**	**60**	**560**

## The current state of pediatric rheumatology in Africa

### A. Research

Epidemiological data concerning PRDs among children across Africa is scarce [20]. There are insufficient resources for research [21–23]. There is considerably less industry support in African countries [24, 25]. Studies from Africa are mostly hospital-based case series and may lack applicability to the community [26].

Despite the challenges, there are deliberate efforts to bridge the research gap in pediatric rheumatology across Africa [22–27]. Research has been carried out in the field of lupus and juvenile idiopathic arthritis among other rheumatic diseases [1, 11, 19–27]. It is imperative that we not only describe the disease phenotypes but aim to include our patients in biological research.

In Kenya and South Africa for example, there are nationwide collaborative studies on Multisystem Inflammatory Syndrome in Children (MIS-C) in order to describe the clinico-epidemiological characteristics and outcomes of MIS-C [28]. The African League Against Rheumatism (AFLAR) study on COVID-19 showed the benefits of pulling together both adult and pediatric rheumatologists to work on a common research agenda for the continent [29]. This is an indicator of the potential for more research collaborations among member countries of the AFLAR involving both pediatric and adult rheumatologists [29].

One of the key pillars in PAFLAR’s strategic plan is to build research by promoting collaborative efforts, establishment of registries, an ethical review board and a journal to showcase research from Africa. This shall be spearheaded by the existing PAFLAR Scientific Committee.

### B. Models of Care

#### Telemedicine

There is a paucity of medical personnel across Africa with only 2.7 physicians per 10,000 people in Africa compared to 5.9 in South East Asia, 12.7 in the eastern Mediterranean, 15.5 in the Western Pacific, 21.5 in the Americas, and 32.1 in the European region [30]. Retention of trained specialists poses a challenge in Africa due to a lack of supportive frameworks and ‘brain drain’ [31]. This is challenging particularly when health systems are strained. Telemedicine offers a plausible solution [32]. Recently, there has been increased access to and reliance on remote meeting systems [33]. Tele-consults through platforms such as zoom can be used to enhance multi-disciplinary care for patients. Pediatric rheumatology teams in Nigeria and Kenya for example organize zoom meetings on a need to need basis to discuss and deliberate on patients they mutually have in common in a multi-disciplinary approach. Further research is required to explore how best to utilize telemedicine to promote pediatric rheumatology in Africa. In solidarity with global partners, researchers from Africa are participating in the validation of the virtual pediatric gait, arms, leg, spine (PGALS) screening examination as part of a global telemedicine initiative [33]. More research is needed to explore the feasibility of telemedicine for pediatric rheumatology in Africa.

### C. Education and Training

#### I) PAFLAR Webinars

Since its establishment on September 7th, 2019, the **Pediatric Society of the African League Against Rheumatism (PAFLAR)** embarked on a series of webinars from the 5 regions of Africa i.e. northern, eastern, southern, central and western regions to help bridge the gap in pediatric rheumatology education. This has helped bring the pediatric rheumatology family in Africa much closer allowing exchange of knowledge where distance has been bridged by digital technology. This also serves as a platform to begin academic mentorship of pediatric trainees who log onto the webinars. In order to expand its reach, PAFLAR shall be collaborating with the Juvenile Inflammatory Rheumatism (JIR) winter school in Switzerland through facilitation of an ILAR grant to offer virtual conferences and webinars to members of both PAFLAR and JIR. The goal is to offer a blended learning experience by combining our PAFLAR webinars and the JIR rheumatology courses through fee subsidies to enrich the learning experience of pediatric rheumatologists and all other healthcare workers who care for pediatric rheumatology patients in the African continent while offering them an opportunity to showcase their clinical experience and expertise with the global rheumatology community.

#### II) Pediatric Rheumatology Training

There have been various initiatives to help bridge the gap in the pediatric rheumatology workforce [34–37]. This includes the **UWEZO project**, a collaboration between Kenyan, United Kingdom (UK) and Swedish rheumatologists who trained an estimated 500 physicians and health workers at 11 sites across Kenya [38]. The International League of Associations for Rheumatology (ILAR) supported onsite training in Zambia for 2 years under **the EPAREP project** (Enhancement of Pediatric and Adult Rheumatology Education and Practice) [39].

In early 2009, the International League of Associations for Rheumatology (ILAR) funded a program known as the **“East Africa Initiative”** in order to unite the international rheumatology community to promote rheumatology services in an area that carries 25% of the world’s disease burden but has only 2% of the world’s human resources for health [40]. Consequently, training for the only 2 pediatric rheumatologists in East and Central Africa, who are based in Kenya, was supported by rheumatology units in the United Kingdom (UK,) Canada and South Africa. Collaborations have spurred the growth of pediatric rheumatology in Africa through foundations such as **“Rheumatology for All”** with outreach activities in Ethiopia and Rwanda (https://rheumatologyforall.org).

Developing hybrid programs of local and international training, as occurs in Kuwait and Saudi Arabia, may be more feasible and sustainable [14, 15]. The Pediatric Rheumatology European Society (PReS) and other bodies may look to set up **“sister hospital initiatives”** where **“areas of need”** partner with well-resourced hospitals to provide education and clinical support. A similar initiative was successfully undertaken as part of the southern hemisphere educational partnership for pediatric arthritis and rheumatological diseases **(SHEPPARD**) program between Argentina and South Africa [34]. These kinds of activities happen informally all the time, but a centralized or regional system would enable people to access them more easily.

## Conclusion

This is a long journey which has already started and is gaining momentum. Initiatives such as the creation of a Pediatric Society of the African League Against Rheumatism **(PAFLAR)**, the creation of Global Task Force for Musculoskeletal Health and **PReS** (Pediatric Rheumatology European Society) initiatives indicate that a core group of rheumatology health care providers and indeed the global community, have recognized that reaching out to the millions of children who live with rheumatic diseases in areas or situations where appropriate care is unavailable or inaccessible is a moral imperative.

## Data Availability

Not Applicable.
